# Stokes–Mueller polarization-based analysis of model SARS-CoV-2 virions

**DOI:** 10.1007/s10103-022-03680-3

**Published:** 2023-01-09

**Authors:** Spandana K U, Bhagesh Basavraj Hunakunti, Aymeric Le Gratiet, Ankur Gogoi, Nirmal Mazumder

**Affiliations:** 1 Department of Biophysics, Manipal School of Life Sciences, Manipal Academy of Higher Education, Manipal, Karnataka India 576104; 2 Université de Rennes, CNRS, Institut FOTON - UMR 6082, F-22305 Lannion, France; 3 Department of Physics, Jagannath Barooah College, Jorhat, 785001 Assam India

**Keywords:** SARS-CoV-2, Discrete dipole approximation, Mueller matrix, Stokes vector, Polarimetry, Lu-Chipman decomposition

## Abstract

Understanding the virology of the coronavirus at the structural level has gained utmost importance to overcome the constant and long-term health complications induced by them. In this work, the light scattering properties of SARS-CoV-2 of size 140 nm were simulated by using discrete dipole approximation (DDA) for two incident wavelengths 200 nm and 350 nm, respectively. Three different 3-dimensional (3D) models of SARS-CoV-2 corresponding to 15, 20, and 40 numbers of spike proteins on the viral capsid surface were constructed as target geometries for the DDA calculations. These models were assessed by employing Stokes–Mueller polarimetry to obtain individual polarization properties such as degree of polarization (DOP), degree of linear polarization (DOLP), and degree of circular polarization (DOCP). Irrespective of its spike numbers, all the coronavirus models were found to display higher DOP and DOCP values and negligibly small DOLP values for circularly polarized incident light, indicating the presence of chiral structures. On the other hand, the lack of understanding about the dependence of the Mueller matrix on its microstructural properties was overcome by transforming 16 Mueller elements into sub-matrices with specific structural and physical properties using Lu–Chipman-based Mueller matrix polar decomposition method. The obtained properties such as retardance, diattenuation, and depolarization were used for investigating the composition and microstructural information. The approach presented in this work has the potential to understand the virology of the coronavirus at the structural level and, therefore, will be beneficial in developing effective detection strategies by exploiting their characteristic electromagnetic scattering signatures.

**Supplementary Information:**

The online version contains supplementary material available at 10.1007/s10103-022-03680-3.

## Introduction

The ongoing corona pandemic crisis is caused by an infection of severe acute respiratory syndrome-related coronavirus-2 (SARS-CoV-2), leading to inflammatory conditions in human lungs. An evolved beta-corona virus similar to human COVID-19 causing SARS-CoV-2 in *Rhinolophus sinicus* (horseshoe bats) [1] was discovered in China soon after the first transmission emergence of SARS-CoV from animals to humans [2]. Epidemiologically, pneumonia incidents in human hosts are a result of varied viral strains, which include adenovirus, influenza virus, Middle East respiratory syndrome virus (MERS-V), parainfluenza virus, respiratory syncytial virus (RSV), SARS-CoV, and enteric enveloped CoV [3–6]. On 13^th^ January 2020, The World Health Organization (WHO) coined the official terminology 2019-Novel Coronavirus (2019-nCoV), and simultaneously, the disease caused by 2019-nCoV was termed as Coronavirus Disease-2019 (COVID-19) on 11^th^ February 2020 [7]. Due to its similarity with the novel coronavirus and SARS-CoV, the International Committee on Taxonomy of Viruses (ICTV) declared the official nomenclature of the virus as SARS-CoV-2 [8]. SARS-CoV-2 has resulted in unforeseen public health and economic threats worldwide [9]. Globally, as of 25^th^ January 2022, there have been 352,796,704 confirmed cases of COVID-19, including 5,600,434 deaths, reported to WHO [10]. The effect of COVID-19 on humankind has been devastating that the global stock markets experienced their worst crash since 1987, and in the first three months of 2020, the G20 economies fell 3.4% year-on-year [11].

Remarkably, with a size of 60–140 nm and constituting a ( +) ssRNA genome of 29,891 bp in size (functions as mRNA directly which encodes 9860 amino acids), SARS-CoV-2 can divide faster and infect rapidly, causing a global health-threatening emergency [12]. Structurally, SARS-CoV-2 constitutes a spike (S) glycoprotein, dimeric HE enzyme, a membrane matrix glycoprotein (M), and RNA [13]. The main point of contact with the host cell is mediated by the S glycoprotein directly and indirectly in the infection cycle. A receptor-binding domain (RBD) is borne on the S glycoprotein of all coronaviruses. The binding of S glycoprotein RBD to its host receptor causes cleavage of this glycoprotein by a host furin-like protease, which releases the spike fusion peptides, thus facilitating entry into the host cell [14]. S glycoprotein has been found to have numerous binding and neutralization epitopes, making it an essential target for vaccine design [15–17]. Notably, even before WHO declared a worldwide pandemic, researchers have been on their toes to develop anti-viral drugs against SARS-CoV-2. Nevertheless, coronavirus has been successful in expanding their host ranges, including humans, to their recombination, mutator alleles, and mutational/evolutionary mechanisms [18]. Thus, it has become crucial to understand the virology of the coronavirus at a structural level due to constant as well as long-term health complications from these zoonotic viruses [19].

In this context, polarized light scattering characteristics of particulate matter, including biological entities, carry a lot of information about their physical and optical properties [20]. Notably, the 4 × 4 Mueller matrix connects the Stokes vectors of the light incident on and scattered by the system of particles under study. The Mueller matrix, which depends on several parameters related to the scattering particle, such as size, shape, and refractive index, can be utilized to extract some of the physical and optical characteristics of the scatterer. In particular, the sample assessment by using Mueller matrix polar decomposition method distinguishes multiply scattered light, revealing individual polarization properties such as retardance, diattenuation, and depolarization [20], which can further be used to extract their compositional and microstructural information. Similarly, approaches based on Stokes–Mueller formalism could be extremely beneficial in understanding the virology of the coronavirus at a structural level. Several studies have been carried out in the past to characterize coronavirus and other virus particles by using light scattering techniques [21–25]. Recently, Petrov calculated the scattering properties of coronavirus particles by using spherical model particles with various spike protein numbers. This work was primarily focused to study the effect of spike proteins on the scattered intensity and degree of linear polarization [26]. On the other hand, the study by Ashraf et al. [27] emphasized the information embedded in the circular polarization state of the scattered light to detect the presence of genome in the virus particle. The group showed that the circular intensity differential scattering is sensitive to the number of turns, handedness, and diameter of the RNA genome. These results greatly improved our understanding of interactions between coronavirus with electromagnetic radiations, whilst using only one or two of the 16 Mueller matrix (MM) elements. Remarkably, it is possible to extract additional optical parameters characteristic to the scattering particle by utilizing other MM elements so that the scatterer of interest (coronavirus in this case) can be properly discriminated from other scattering particles. In this work, Stokes vectors and various polarization parameters of the light scattered from various coronavirus models with different spike numbers are calculated at 200 nm and 350 nm incident wavelengths. Further, Lu–Chipman-based Mueller matrix polar decomposition method is employed in this work to investigate the interaction of coronavirus models with different spike numbers [28] and understand a complete set of physical parameters resulting from the decomposition.

## Materials and methods

### Stokes–Mueller formalism

Polarization is a fundamental property of electromagnetic radiation (light), and the interactions of polarized light with biological samples can reveal structural information associated with its pathological condition. Even the slightest variation in structural alignment can induce a significant change in polarization property, which can play a crucial role in the early detection of abnormal morphology [29]. In this regard, Sir George Gabriel represented polarization behavior in terms of observables and described the complete state of polarization of light in terms of four measurable quantities, known as the Stokes parameters. Total optical field intensity is described by the first parameter, and the remaining parameters describe the polarization state. The Stokes parameters are a logical consequence of wave theory and can be arranged in a 4 × 1 column matrix as

1 $$\mathrm S=\begin{bmatrix}{\mathrm S}_0\\{\mathrm S}_1\\{\mathrm S}_2\\{\mathrm S}_3\end{bmatrix}=\begin{bmatrix}{\mathrm E}_{\mathrm{ox}}^2+{\mathrm E}_{\mathrm{oy}}^2\\{\mathrm E}_{\mathrm{ox}}^2-{\mathrm E}_{\mathrm{oy}}^2\\2{\mathrm E}_{\mathrm{ox}}{\mathrm E}_{\mathrm{oy}}\mathrm{cos \delta}\\2{\mathrm E}_{\mathrm{ox}}{\mathrm E}_{\mathrm{oy}}\mathrm{sin \delta}\end{bmatrix}=\begin{bmatrix}{\mathrm I}_0+{\mathrm I}_{90}\\{\mathrm I}_0-{\mathrm I}_{90}\\{\mathrm I}_{45}-{\mathrm I}_{135}\\{\mathrm I}_{\mathrm R}-{\mathrm I}_{\mathrm L}\end{bmatrix}$$

where *S*_0_ represents the total light intensity, *S*_1_ is the difference between 0° and 90° polarization intensities, *S*_2_ is the difference between + 45° and − 45° (135°) polarization intensities, and *S*_3_ is a difference between the left and right circular polarization intensities [30]. Several polarization parameters such as degree of polarization (DOP), degree of linear polarization (DOLP), and degree of circular polarization (DOCP) can be constructed from the Stokes parameters as follows,

2 $$\mathrm{DOP}=\frac{\sqrt{{\mathrm S}_1^2+{\mathrm S}_2^2+{\mathrm S}_3^2}}{{\mathrm S}_0}$$

3 $$\mathrm{DOLP}=\frac{\sqrt{{\mathrm S}_1^2+{\mathrm S}_2^2}}{{\mathrm S}_0}$$

4 $$\mathrm{DOCP}=\frac{\left|{\mathrm S}_3\right|}{{\mathrm S}_0}$$

In general, fully polarized light gets depolarized when it transmits through a scattering environment [31, 32]. While DOP of a fully polarized light is 1 and is 0 for unpolarized light, DOP for a partially polarized light is between 0 and 1. Thus, the DOP of the scattered light signifies the amount of incident polarization that persists in the scattered light. Similarly, the DOLP quantitively describes the amount of linear polarization present in the light beam and essentially illustrates the anisotropy of the distribution of molecular alignment/orientation in the scattering volume. DOCP, on the other hand, is a measure of how effectively chiral molecules interact with the circularly polarized light. The values of both DOLP and DOCP ranges from 0 to 1 [33, 34]. Although the Stokes parameters describe the polarization properties of light, Stokes–Mueller formalism is suitable for detailed analysis of the sample. The transformation of polarization property of light due to its interaction with an optical system can be described by Mueller matrix and characterized by Mueller formalism. When Stoke vector, *S*_*i*_ of incoming light propagates through a scattering media, the output Stoke vector, *S*_*i*_*′* can be obtained as a linear combination of the four Stokes parameters of the incident beam in terms of matrix form,

5 $$\left[\begin{array}{c}{\mathrm {S}_{0}}^{^{\prime}}\\ {\mathrm {S}_{1}}^{^{\prime}}\\ {\mathrm {S}_{2}}^{^{\prime}}\\ {\mathrm {S}_{3}}^{^{\prime}}\end{array}\right]= \left[\begin{array}{c}\mathrm {m}_{00\;}\mathrm { m}_{01\;}\mathrm { m}_{02\;}\mathrm {m}_{03}\\ \mathrm {m}_{10\;}\mathrm { m}_{11\;} \mathrm {m}_{12\;}\mathrm { m}_{13}\\ \mathrm {m}_{20\;}\mathrm { m}_{21\;}\mathrm { m}_{22\;}\mathrm {m}_{23}\\ \mathrm {m}_{30\;}\mathrm { m}_{31\;} \mathrm {m}_{32\;}\mathrm {m}_{33}\end{array}\right]\left[\begin{array}{c}\mathrm {S}_{0}\\ \mathrm {S}_{1}\\ \mathrm {S}_{2}\\ \mathrm {S}_{3}\end{array}\right]$$

or

6 $$\mathrm {S}^{^{\prime}}=\mathrm {M}.\mathrm{S}$$

where $$\mathrm M=\begin{bmatrix}{\mathrm m}_{00\;}{\mathrm m}_{01\;}{\mathrm m}_{02\;}{\mathrm m}_{03}\\{\mathrm m}_{10\;}{\mathrm m}_{11\;}{\mathrm m}_{12\;}{\mathrm m}_{13}\\{\mathrm m}_{20\;}{\mathrm m}_{21\;}{\mathrm m}_{22\;}{\mathrm m}_{23}\\{\mathrm m}_{30\;}{\mathrm m}_{31\;}{\mathrm m}_{32\;}{\mathrm m}_{33}\end{bmatrix}$$ is the Mueller matrix.

where *m*_*ij*_ are experimentally measurable quantities, *i*, *j* = 0 to 3.

The Mueller matrix provides a comprehensive explanation of the sample’s optical and structural information related to polarization, thus playing an important role in biomedical research and sample characterization. However, there exists a lack of explicit association of Mueller matrix with microstructural properties. Mueller matrix elements reflect lumped effects due to the simultaneous occurrence of several polarization effects, hence hindering the unique interpretation [35–38]. The Mueller matrix of the analyzed sample encloses its combined polarizing properties. However, the 16 Mueller elements can be transformed into sub-parameters with specific structural and physical properties using Lu–Chipman-based Mueller matrix polar decomposition (MMPD) method. Notably, assessment of biological sample by means of polar decomposition approach, where decomposition of Mueller matrix into three basis matrices can be used as an effective tool to distinguish multiply scattered light and gain individual polarization properties [39]. Polarization properties such as retardance, diattenuation, and depolarization can be used for investigating composition and microstructural information and could become beneficial in understanding the virology of the corona virus at a structural level.

In this work, Mueller matrix representing SARS-COV-2 was decomposed into a set of three basis matrices representing diattenuator, retarder, and depolarizer, respectively. These sub-matrices were converted into individual parameters associated to diattenuation, retardance, and depolarization properties. Mueller matrix “*M*” is decomposed as

$${\mathrm M=\mathrm M}_{\mathrm\Delta}{\mathrm M}_{\mathrm R}{\mathrm M}_{\mathrm D}$$

where depolarization matrix, *M*_Δ_, describes depolarization effect of the medium, a retardance matrix, *M*_*R*_, accounts for the effect of optical activity and linear birefringence, and a diattenuation matrix, *M*_*D*_, involves the effect of circular and linear dichroism. The basis matrices were examined further to extract individual polarization properties of medium such as, optical rotation (*Ψ*), linear retardance (*δ*), and its orientation angle (*θ*), depolarization coefficient (*∆*), and diattenuation (*d*).

The diattenuation matrix, ${M}_{D}$, is defined using a relation

$${\mathrm M}_{\mathrm D}=\begin{bmatrix}1&\overrightarrow{\mathrm d}^{\mathrm T}\\\mathrm d^\rightarrow&{\mathrm m}_{\mathrm D}\end{bmatrix}$$

where ${m}_{D}$ is a 3 × 3 submatrix and the diattenuation vector $\overrightarrow{d}$ is defined as

$$\overrightarrow{\mathrm d}=\left\{1/\mathrm M\right.\left.(0,0)\right\}\times\left[\mathrm M\left(0,1\right)\mathrm M\left(0,2\right)\mathrm M(0,3)\right]^{\mathrm T}$$

The magnitude of diattenuation was determined using a relation

$$\mathrm{d}=\left\{1/\right.\left. \mathrm M(\mathrm{0,0})\right\}\times {\left.\left[{\left\{\mathrm M\right.\left.(\mathrm{0,1})\right\}}^{2}\right.+{\left\{\mathrm M\right.\left.(\mathrm{0,2})\right\}}^{2}+{\left\{\mathrm M\right.\left.(\mathrm{0,3})\right\}}^{2}\right]}^{{~}^{1}\!\left/ \!{~}_{2}\right.}$$

The elements of the Mueller matrix, *M*, are represented by the *M*(*i,j*).

The depolarization matrix, *M*_*Δ*_, is defined as

$${\mathrm M}_{\mathrm\Delta}=\begin{bmatrix}1&\overrightarrow0^{\mathrm T}\\{\overrightarrow{\mathrm P}}_{\mathrm\Delta}&{\mathrm m}_{\mathrm\Delta}\end{bmatrix}$$

Here, *m*_*Δ*_ is the 3 $\times$ 3 depolarization submatrix. The parameter *P*_Δ_ depends on diattenuation (*d*) and polarization (*P*) [39]. The depolarization coefficients were calculated from the diagonal elements of the depolarization matrix *M*_*Δ*_. The net depolarization coefficient, *Δ*, is measured using a relation:

$$\mathrm\Delta=1-\left.\left\{\left|\mathrm{tr}({\mathrm M}_{\mathrm\Delta}-1\right|\right./3\right\}$$

The retardance matrix *M*_*R*_ was obtained using the relation

$${\mathrm M}_{\mathrm R}=\begin{bmatrix}1&\overrightarrow0^{\mathrm T}\\\overrightarrow0&{\mathrm m}_{\mathrm R}\end{bmatrix}$$

The total retardance, *R* (the coupled effect of circular and linear birefringence), was determined from retardance matrix, *M*_*R*_, using the relationship:

$$\mathrm R=\cos^{-1}\left\{\frac{\mathrm{tr}({\mathrm M}_{\mathrm R})}2-1\right\}$$

**Fig. 1:**
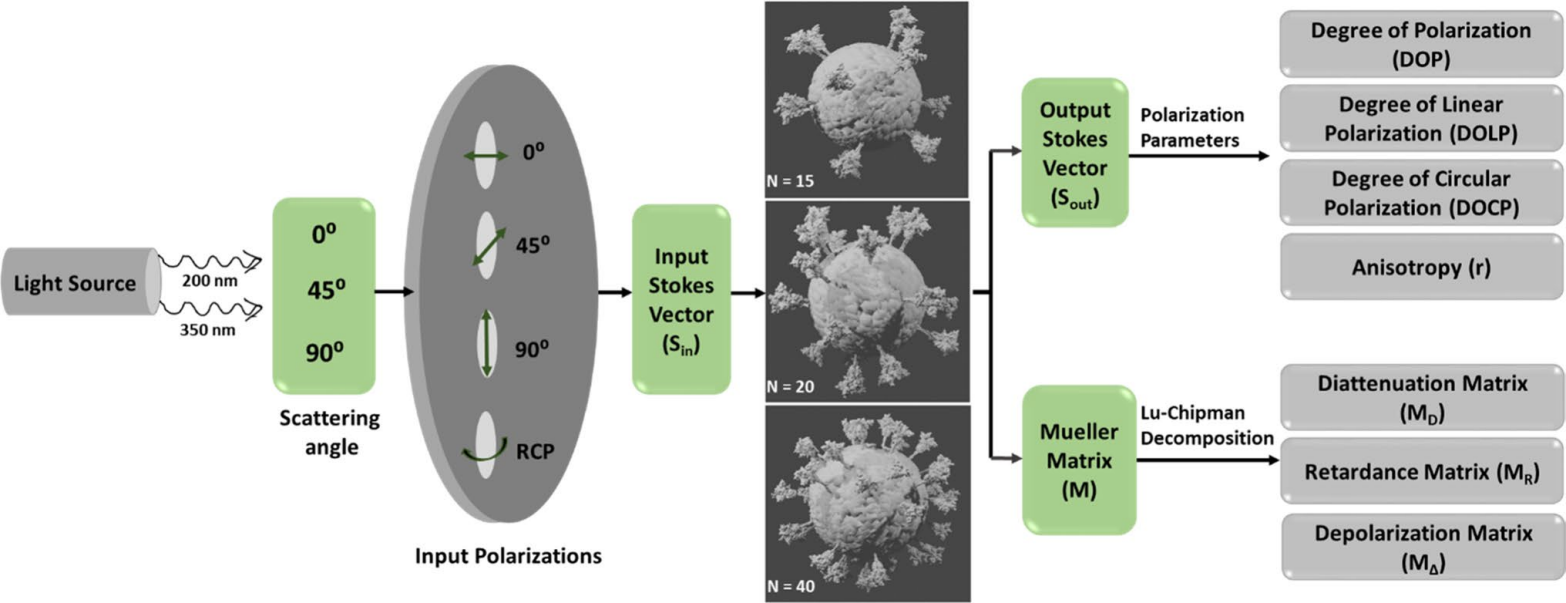
Schematic representation of Stokes–Mueller analysis with various SARS-CoV-2 models

The total retardance matrix, *M*_*R*_, was denoted as a matrix combination for a circular retarder (optical rotation with magnitude of *ψ*) and linear retarder (having a magnitude of linear retardance = *δ*). The schematic representation of the Stokes–Mueller analysis is shown in Fig. 1.

### Modeling SARS-CoV-2

**Fig. 2:**
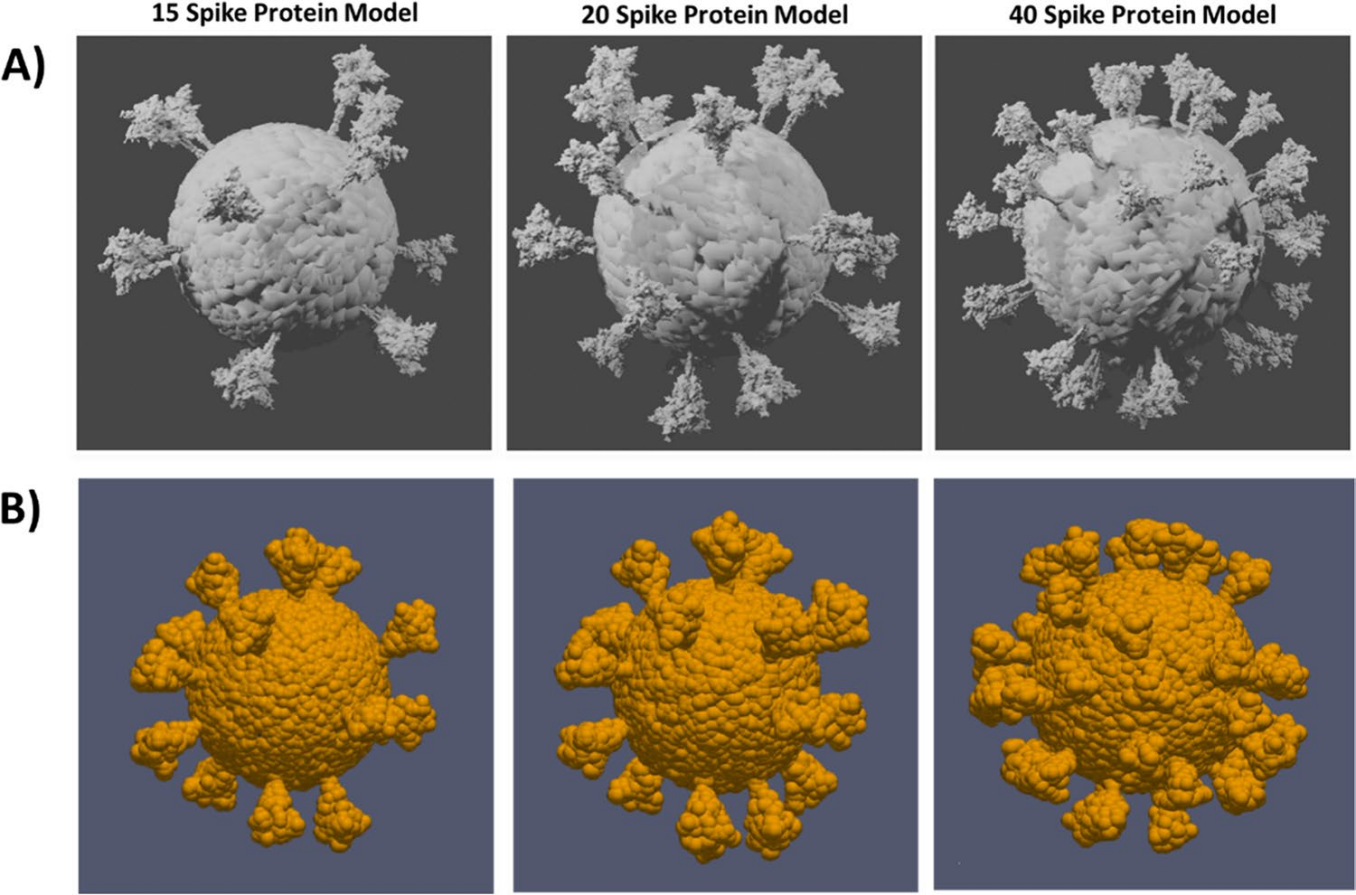
(**A**) The blender representation of the target shapes of SARS-COV-2 with different spike numbers used in DDA computations. Model references: PDB ID 6VXX, CHARMM-GUI Archive-COVID-19 Proteins Library. (**B**) 3D rendered dipole representation of the target geometries (SARS-CoV-2) with 15, 20, and 40 spike proteins

In this work, light scattering properties of SARS-CoV-2 were simulated in terms of angle-resolved Mueller matrix elements for two incident wavelengths (200 nm and 350 nm) by using DDSCAT 7.3.0 [40–42] based on discrete dipole approximation (DDA). Wavelengths higher than these wavelengths (e.g., 500 nm and 1100 nm) are not reported here since at such larger wavelengths the contributions from the spikes to the overall scattering of a virion became very weak that no noticeable variation in the scattering properties could be observed as compared to a solid sphere of same size and refractive index. Notably, DDA (also referred to as the coupled dipole method) is one of the most widely used methods for calculating electromagnetic scattering properties of arbitrary shaped, inhomogeneous, anisotropic, and optically active particles [40, 43]. It employs a volume-based discretization by filling up the target volume with a finite array of polarizable points or dipoles. The interaction of these dipoles with the incident electric field and each other results in the total secondary scattered radiation. In essence, DDSCAT is the numerical implementation of DDA in the form of a freely available open-source Fortran-90 software package developed by Bruce T. Draine and Piotr J. Flatau [41, 42]. In addition to a variety of pre-defined standard and regular target geometries (e.g., cylinders, ellipsoids, and hexagonal prisms), the DDSCAT can also import DDA compatible user defined arbitrary target geometries for the calculation of their light scattering properties. Here, three different shapes of SARS-CoV corresponding to 15, 20, and 40 numbers of spike proteins on the viral capsid surface were constructed as target geometries for the calculations as shown in Fig. 2A. It was reported that about 40 spikes decorate the virion in a SARS-CoV 2 [44]. Open-source modelling tools Blender (V2.90.1) and Autodesk meshmixer (V3.5.474) were used to construct a SARS-CoV-2 3-dimensional (3D) model. Protein data bank (PDB) structure of the SARS-CoV-2 spike glycoprotein (closed state) [45] along with SARS (2002) Spike S-2P with receptor binding domain (RBD) down in the inactive conformation and SARS-CoV-2 Open Spike (S-Protein) was used as a reference to construct the spike protein. Subsequently, these target geometries were converted into DDSCAT compatible dipole arrays by using the open-source target generation tool DDSCAT Convert (https://nanohub.org/resources/ddaconvert), of which the 3D rendered representations were realized by using ParaView software (https://www.paraview.org/), as shown in Fig. 2B. The converted dipole arrays were subsequently used as the shape files in DDSCAT to calculate the light scattering properties.

### Simulation of light scattering properties of SARS-CoV 2 using discrete dipole approximation (DDA)

The target geometries were represented in the form of dipole arrays consisting of ~ 100 k dipoles for all the three SARS-CoV-2 models with 15, 20, and 40 spikes. These dipole numbers satisfied the so-called $|m|kd$ criterion required for the accurate calculation of Mueller matrix elements [40, 42]. The size of SARS-COV-2 was considered to be 140 nm, and the refractive indices of the virion and the nucleic acid were considered to be 1.48 and 1.68, respectively. The relevant parameters of DDA calculation are given in Table 1. Notably, the calculated light scattering properties were averaged over the angles $\beta$ (from 0° to 360°), $\Theta$ (from 0° to 180°), and $\Phi$ (from 0° to 360°) to account for the random orientation of the target geometries with respect to the scattering plane. However, orientation averaging significantly increased the computation time. Therefore, computations were restricted to 27 target orientations ($\beta$ = 0°, 120°, 240°; $\Theta$ = 0°, 60°, 120°; and $\Phi$ = 0°, 120°, 240°) due to the limitations in computational resources and the enormous time required for light scattering calculations. The graphical representations of the calculated Mueller matrix elements are shown in Supplementary Fig. 1. The results are also compared with Mie calculations for a solid sphere of radius 140 nm and refractive index 1.48, obtained by using the code TUMiescat.c [46]. Remarkably, significant variations in the scattering properties were observed for all the three target geometries as compared to that of the solid sphere, which may be attributed to their structural (surface roughness and presence of spikes in virus geometries) and compositional (refractive indices for virus body and nucleic acid) differences.

**Table 1:** Relevant parameters for DDA calculation

Effective radius (nm)^1^	Complex refractive index		Wave-length (nm)	No of spikes	No of dipoles	Average computation time (in seconds)
	Virion^2^	Nucleic acid^3^				
140	Real part = 1.48 Imaginary part = 0.00	Real part = 1.68 Imaginary part = 0.00	200	15	100 k	1721
				20		1655
				40		1456
			350	15		764
				20		1257
				40		1280

^1^This value has been adopted from the papers of Zhu et al. [47], and Petrov [22]

^2^This value has been adopted from the RI value of Influenza A Virus published by Wang et al. [48]

^3^This value has been adopted from Inagaki et al. [49]

## Results and discussion

**Fig. 3:**
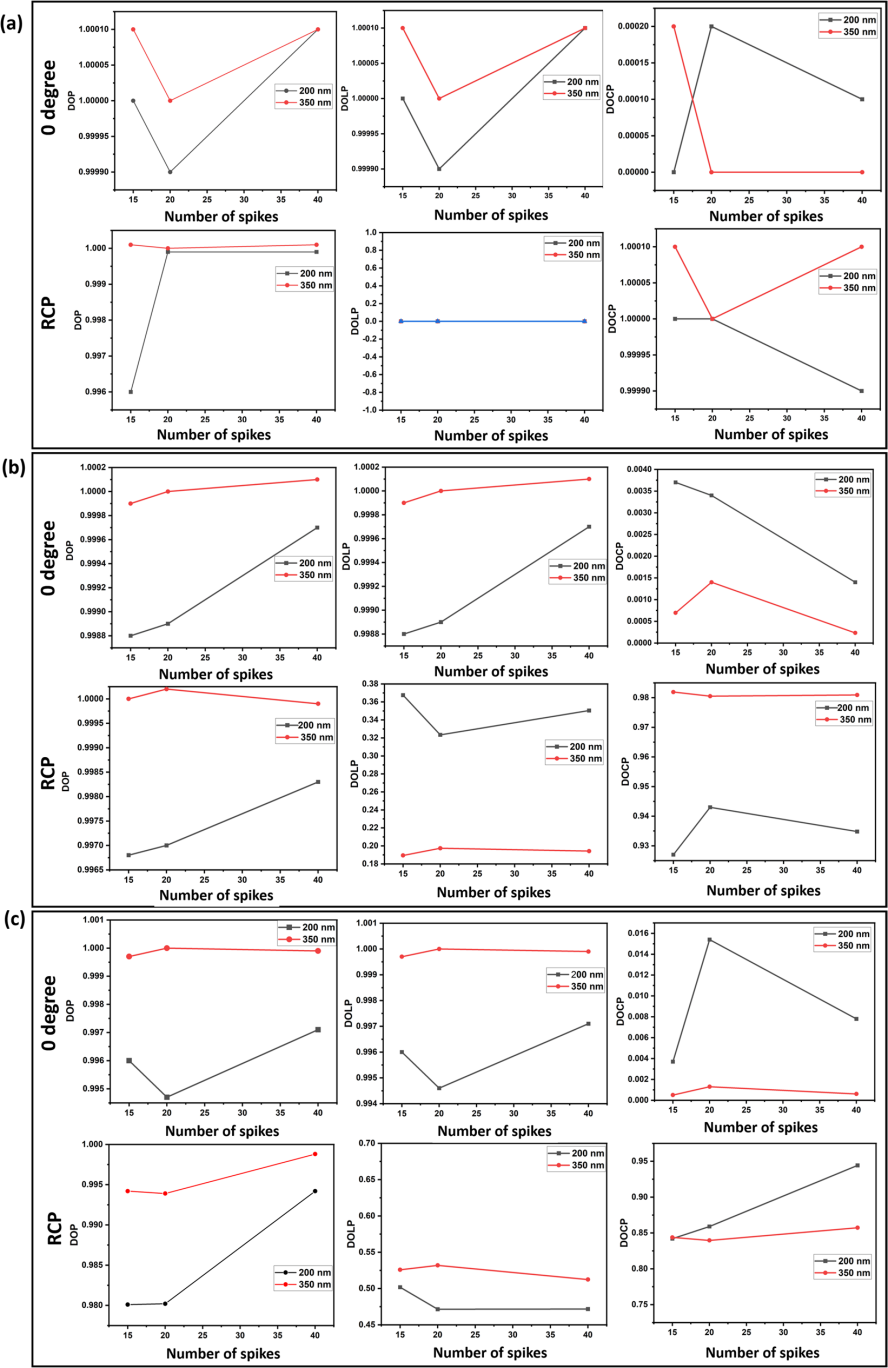
Graphs of Stokes vector polarization parameters such as DOP, DOLP, and DOCP as a function of spike numbers with 0° and RCP excitation polarization states at two incident wavelengths 200 nm and 350 nm for (**a**) 0°, (**b**) 45°, and (**c**) 90° scattering angle


The Mueller matrices of the SARS-CoV-2 models with 15, 20, and 40 spikes were illuminated with light of 0°, 45°, and 90°; subsequently, RCP polarization and output polarization states were determined. The simulation was performed using light of two different wavelengths corresponding to 200 nm and 350 nm. Using MATLAB Software, Stokes vector parameters, *S*_0_, *S*_1_, *S*_2_, and *S*_3_ were reconstructed for both input and output states. The output Stokes vectors and polarization parameters at 0°, 45°, and 90° scattering angles, calculated for these three SARS-CoV-2 models, for various input polarization states are shown in Table 2. For these scattering angles, all the three target geometries were found to exhibit greater values of Stokes polarization parameters (DOP, DOLP, and DOCP) at an incident wavelength of 350 nm than that of 200 nm. Further, for 0° input polarization, the SARS-CoV-2 models display higher DOP and DOLP values and negligibly small DOCP values irrespective of its spike numbers. Remarkably, the models follow reverse trend by exhibiting greater DOP, DOCP values and reduced DOLP values for RCP input. Further, the graph of DOP, DOLP, and DOCP as a function of spike numbers for two different wavelengths, 200 nm and 350 nm, is shown in Fig. 3. The results clearly depict the gradual decrease in DOP and DOLP values with an increase in scattering angle for the coronavirus model with 15 spikes in the case of 0° input polarization, for both 200 nm and 350 nm wavelengths.

**Table 2:** Stokes vectors and various polarization parameters

Spikes	Wavelength	Scatteing angle (*θ*)	Input	*S*_in_	*S*_out_	DOP	DOLP	DOCP
15	200 nm	0°	0°	[1 1 0 0]^T^	[1 1 0.0001 0] ^T^	1	1	0
			45°	[1 0 1 0] ^T^	[1 − 0.0001 1 0] ^T^	1	1	0
			90°	[1 − 1 0 0] ^T^	[1 − 1 0 1] ^T^	1	1	0
			RCP	[1 0 0 1] ^T^	[1 0 0 1] ^T^	1	0	1
		45°	0°	[1 1 0 0]^T^	[1.3387 1.3371 − 0.0024 − 0.0049] ^T^	0.9988	0.9988	3.70E − 03
			45°	[1 0 1 0] ^T^	[0.9998 0.3409 0.9275 0.1348] ^T^	1.1356	1.1276	0.1348
			90°	[1 − 1 0 0] ^T^	[0.6613 − 0.66 0.0009 0.0008] ^T^	0.9988	0.998	0.0012
			RCP	[1 0 0 1] ^T^	[0.9989 0.3404 − 0.1375 0.9256] ^T^	0.9968	0.3675	9.27E − 01
		90°	0°	[1 1 0 0]^T^	[1.4158 1.41 0.0215 − 0.0052] ^T^	0.996	0.996	3.7E − 03
			45°	[1 0 1 0] ^T^	[1.0153 0.4169 0.8551 0.2878] ^T^	0.9789	0.937	0.2835
			90°	[1 -100] ^T^	[0.5842 − 0.579 0.0099 − 0.0044] ^T^	0.9913	0.9912	0.0075
			RCP	[1 0 0 1] ^T^	[0.9928 0.4145 − 0.2763 0.8359] ^T^	0.9801	0.5018	0.842
	350 nm	0°	0°	[1 1 0 0]^T^	[1 1.0001 0 − 0.0002] ^T^	1.0001	1.0001	2E − 04
			0°	[1 1 0 0] ^T^	[1 0 1.0001 0] ^T^	1.0001	1.0001	0
			45°	[1 0 1 0] ^T^	[1 − 1.0001 0 0] ^T^	1.0001	1.0001	0
			RCP	[1 0 0 1] ^T^	[1 0 0 1.0001]	1.0001	0	1.0001
		45°	0°	[1 1 0 0] ^T^	[0.8627 0.8626 − 0.0001 − 0.0006] ^T^	0.9999	0.9999	6.95E − 04
			45°	[1 0 1 0] ^T^	[1.0001 − 0.1373 0.982 − 0.1308] ^T^	1	0.9915	1.31E − 01
			90°	[1 -100] ^T^	[1.1373 − 1.1374 0.0004 0.0005] ^T^	1.0001	1.0001	4.40E − 04
			RCP	[1 0 0 1] ^T^	[0.9999 − 0.1368 0.1309 0.9818] ^T^	1	0.1894	0.9819
		90°	0°	[1 1 0 0] ^T^	[1.4882 1.4877 0.0022 − 0.0008] ^T^	0.9997	0.9997	5E − 04
			45°	[1 0 1 0] ^T^	[1.0038 0.4904 0.8475 − 0.1908] ^T^	0.9938	0.9755	0.1901
			90°	[1 − 1 0 0] ^T^	[0.5118 − 0.5114 0.0037 0.001] ^T^	0.9992	0.9992	2E − 03
			RCP	[1 0 0 1] ^T^	[1.0011 0.4894 0.1939 0.8447] ^T^	0.9942	0.5258	0.8438
20	200 nm	0°	0^**0**^	[1 1 0 0]^T^	[1 0.9999 0 − 0.0002] ^T^	0.9999	0.9999	2E − 04
			45°	[1 0 1 0] ^T^	[1 0 0.9999 − 0.0002] ^T^	0.9999	0.9999	2E − 04
			90°	[1 − 1 0 0] ^T^	[1 − 0.9999 0 − 0.0002] ^T^	0.9999	0.9999	2E − 04
			RCP	[1 0 0 1] ^T^	[0.9998 0 0 0.9997] ^T^	0.9999	0	1
		45°	0°	[1 1 0 0] ^T^	[1.3093 1.3079 − 0.0019 − 0.0045] ^T^	0.9989	0.9989	3.4E − 03
			45°	[1 0 1 0] ^T^	[0.9956 0.3956 0.941 0.0835] ^T^	1.0287	1.0253	0.0839
			90°	[1 − 1 0 0] ^T^	[0.6907 -0.6896 − 0.0057 − 0.0012] ^T^	0.9984	0.9984	0.0017
			RCP	[1 0 0 1] ^T^	[0.998 0.31 − 0.0898 0.9412] ^T^	0.997	0.3234	9.43E − 01
		90°	0°	[1 1 0 0] ^T^	[1.4318 1.4239 0.0216 − 0.022] ^T^	0.9947	0.9946	1.54E − 02
			45°	[1 0 1 0] ^T^	[1.0058 0.5947 0.8723 0.1518] ^T^	1.0604	1.0496	0.1509
			90°	[1 − 1 0 0] ^T^	[0.5682 − 0.5614 − 0.0001 − 0.0011] ^T^	0.988	0.988	0.0019
			RCP	[1 0 0 1] ^T^	[0.9917 0.4421 − 0.1519 0.8523] ^T^	0.9802	0.4714	8.59E − 01
	350 nm	0°	0°	[1 1 0 0]^T^	[1 1 0 0] ^T^	1	1	0
			45°	[1 0 1 0] ^T^	[1 0 1 0] ^T^	1	1	0
			90°	[1 − 1 0 0] ^T^	[1 − 1 0 0] ^T^	1	1	0
			RCP	[1 0 0 1] ^T^	[1 0 0 1] ^T^	1	0	1
		45°	0°	[1 1 0 0] ^T^	[0.8573 0.8573 0.0004 0.0012] ^T^	1	1	1.4E − 03
			45°	[1 0 1 0] ^T^	[0.9998 − 0.2781 0.9804 − 0.1354] ^T^	1.0282	1.0193	1.35E − 01
			90°	[1 − 1 0 0] ^T^	[1.1427 − 1.1427 − 0.0009 − 0.0011] ^T^	1	1	9.62E − 04
			RCP	[1 0 0 1] ^T^	[0.9999 0 0 − 0.9986] ^T^	1.0002	0.1974	0.9805
		90°	0°	[1 1 0 0] ^T^	[1.4827 1.4827 − 0.00024 − 0.002] ^T^	1	1	1.E − 03
			45°	[1 0 1 0] ^T^	[0.9941 0.2603 0.8303 − 0.2203] ^T^	0.9085	0.8811	0.2216
			90°	[1 − 1 0 0] ^T^	[0.5173 − 0.5173 − 0.0054 0.0063] ^T^	1.0001	1.0001	1.22E − 02
			RCP	[1 0 0 1] ^T^	[1.0032 0.4868 0.2185 0.8423] ^T^	0.9939	0.5319	0.8396
40	200 nm	0°	0°	[1 1 0 0]^T^	[1 1.0001 − 0.0001 0.0001] ^T^	1.0001	1.0001	1E − 04
			45°	[1 0 1 0] ^T^	[1 0 1.0001 0.0001] ^T^	1.0001	1.0001	1E − 04
			90°	[1 − 1 0 0] ^T^	[1 − 1.0001 0.0001 0.0001] ^T^	1.0001	1.0001	1E − 04
			RCP	[1 0 0 1] ^T^	[1 0 0 0.9999] ^T^	0.9999	0	0.9999
		45°	0°	[1 1 0 0] ^T^	[1.3285 1.3281 0.0005 − 0.0019] ^T^	0.9997	0.9997	1.4E − 03
			45°	[1 0 1 0] ^T^	[0.9996 0.4445 0.9364 0.1163] ^T^	1.0435	1.037	1.16E − 01
			90°	[1 − 1 0 0] ^T^	[0.6715 − 0.6712 0.0009 0.0023] ^T^	0.9996	0.9996	3.40E − 03
			RCP	[1 0 0 1] ^T^	[1.0021 0.3313 − 0.1163 0.9368] ^T^	0.9983	0.3504	0.9348
		90°	0°	[1 1 0 0] ^T^	[1.3873 1.3832 − 0.0167 0.0108] ^T^	0.9971	0.9971	7.8E − 03
			45°	[1 0 1 0] ^T^	[0.989 0.6566 0.8632 0.2732] ^T^	1.1309	1.0966	0.2762
			90°	[1 − 1 0 0] ^T^	[0.6127 − 0.6112 − 0.0058 − 0.0057] ^T^	0.9976	0.9976	0.0093
			RCP	[1 0 0 1] ^T^	[1.0014 0.3792 − 0.2817 0.8764] ^T^	0.9942	0.4717	0.9942
	350 nm	0°	0°	[1 1 0 0]^T^	[1 1.0001 0 0] ^T^	1.0001	1.0001	0
			45°	[1 0 1 0] ^T^	[1 0 1.0001 0] ^T^	1.0001	1.0001	0
			90°	[1 − 1 0 0] ^T^	[1 − 1.0001 0 0] ^T^	1.0001	1.0001	0
			RCP	[1 0 0 1] ^T^	[1 0 0 − 1.0001] ^T^	1.0001	0	1.0001
		45°	0°	[1 1 0 0] ^T^	[0.8586 0.8587 0.0001 − 0.0002] ^T^	1.0001	1.0001	2.33E − 04
			45°	[1 0 1 0] ^T^	[1.0002 − 0.2746 0.9811 − 0.1333] ^T^	1.0273	1.0186	0.1333
			90°	[1 − 1 0 0] ^T^	[1.1414 − 1.1413 0.0002 − 0.0002] ^T^	0.9999	0.9999	1.75E − 04
			RCP	[1 0 0 1] ^T^	[1 − 0.1411 0.1334 0.9809] ^T^	0.9999	0.1942	0.9809
		90°	0°	[1 1 0 0] ^T^	[1.4813 1.4811 − 0.0008 0.0009] ^T^	0.9999	0.9999	6.07E − 04
			45°	[1 0 1 0] ^T^	[1 0.3045 0.8572 − 0.1761] ^T^	0.9266	0.9097	0.1761
			90°	[1 − 1 0 0] ^T^	[0.5187 − 0.5814 0.0005 0.0006] ^T^	0.9994	0.9994	1.20E − 03
			RCP	[1 0 0 1] ^T^	[1.0007 0.4814 0.1767 0.8579] ^T^	0.9988	0.5124	0.8573

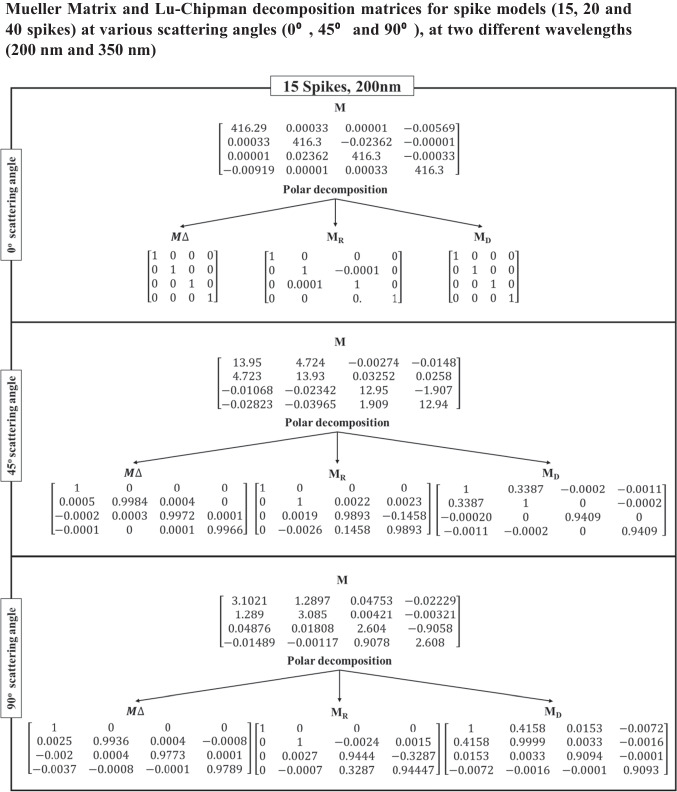
 
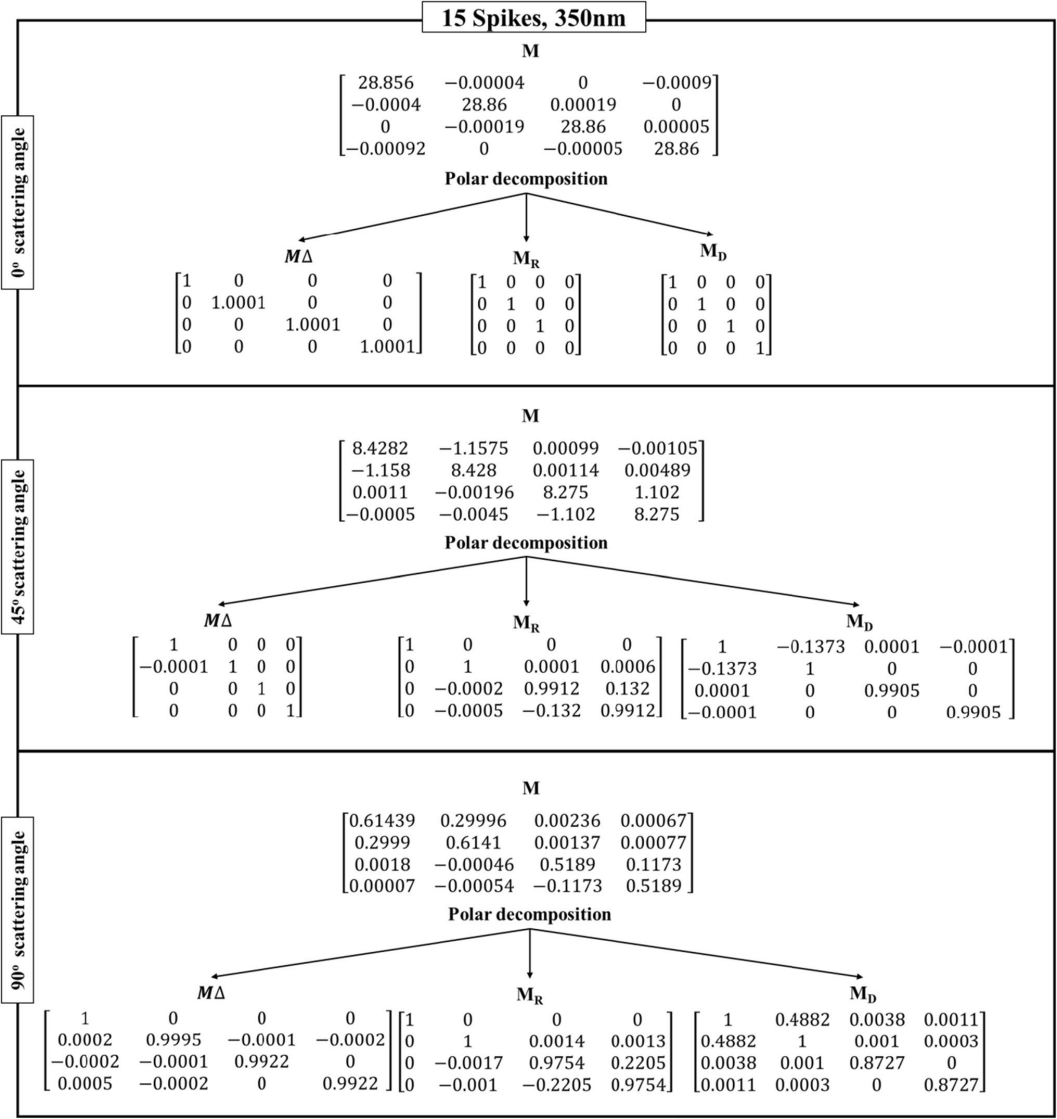
 
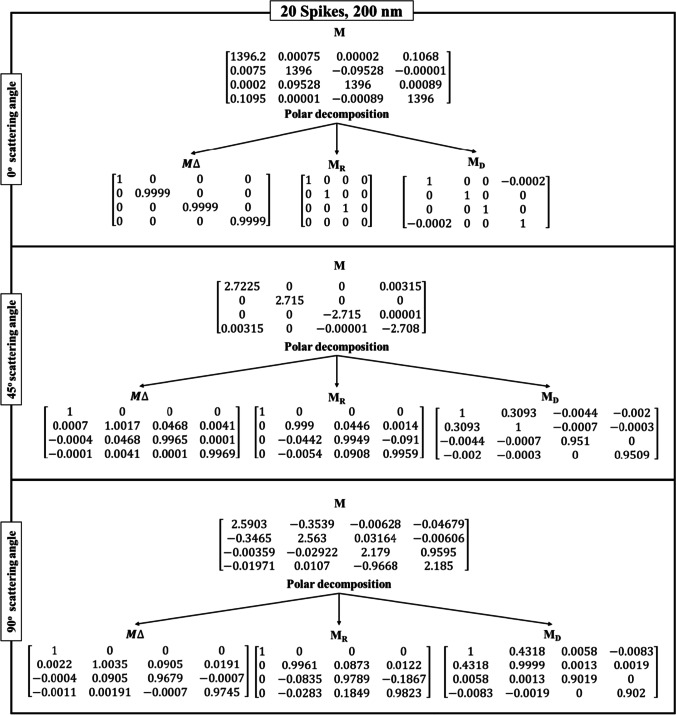
 
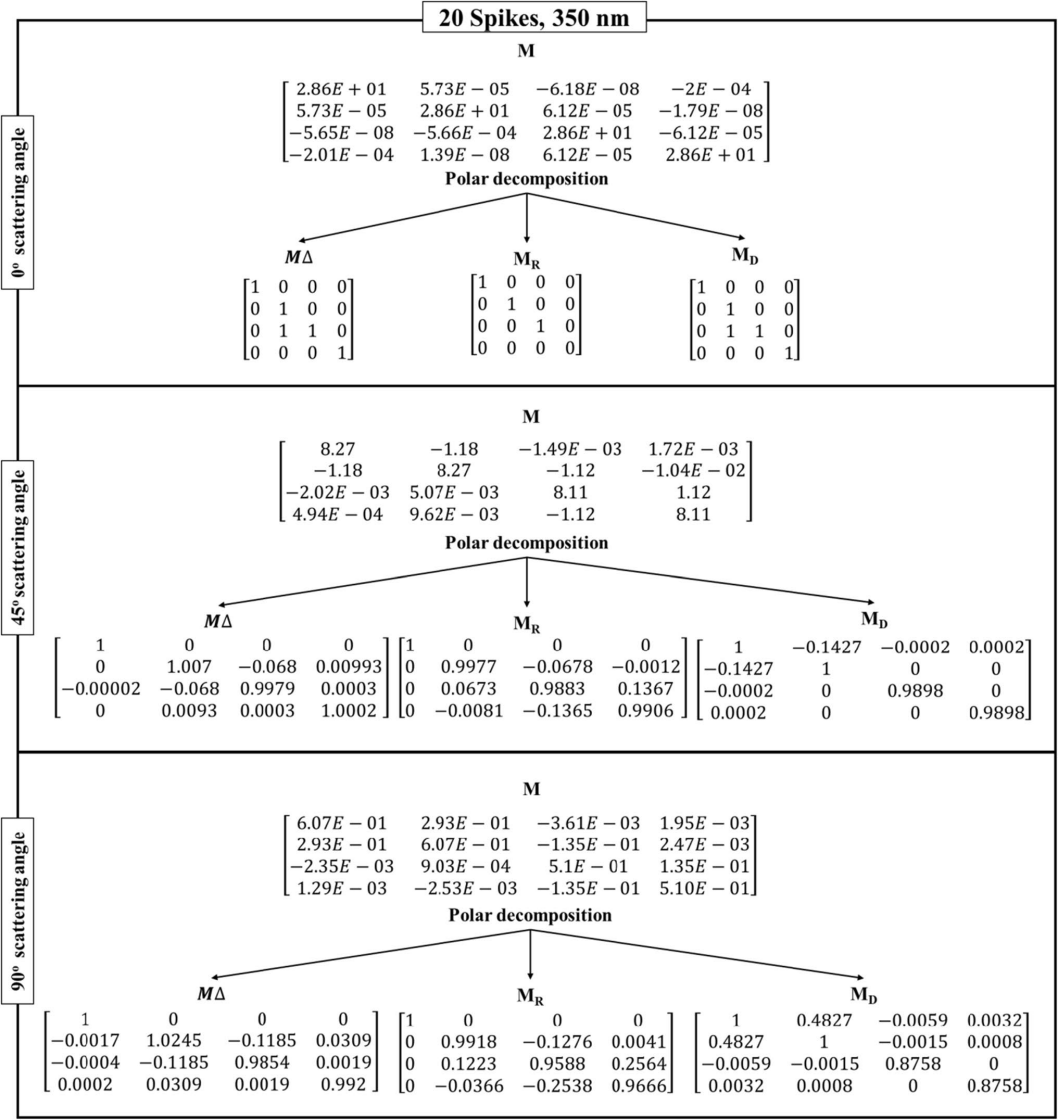
 
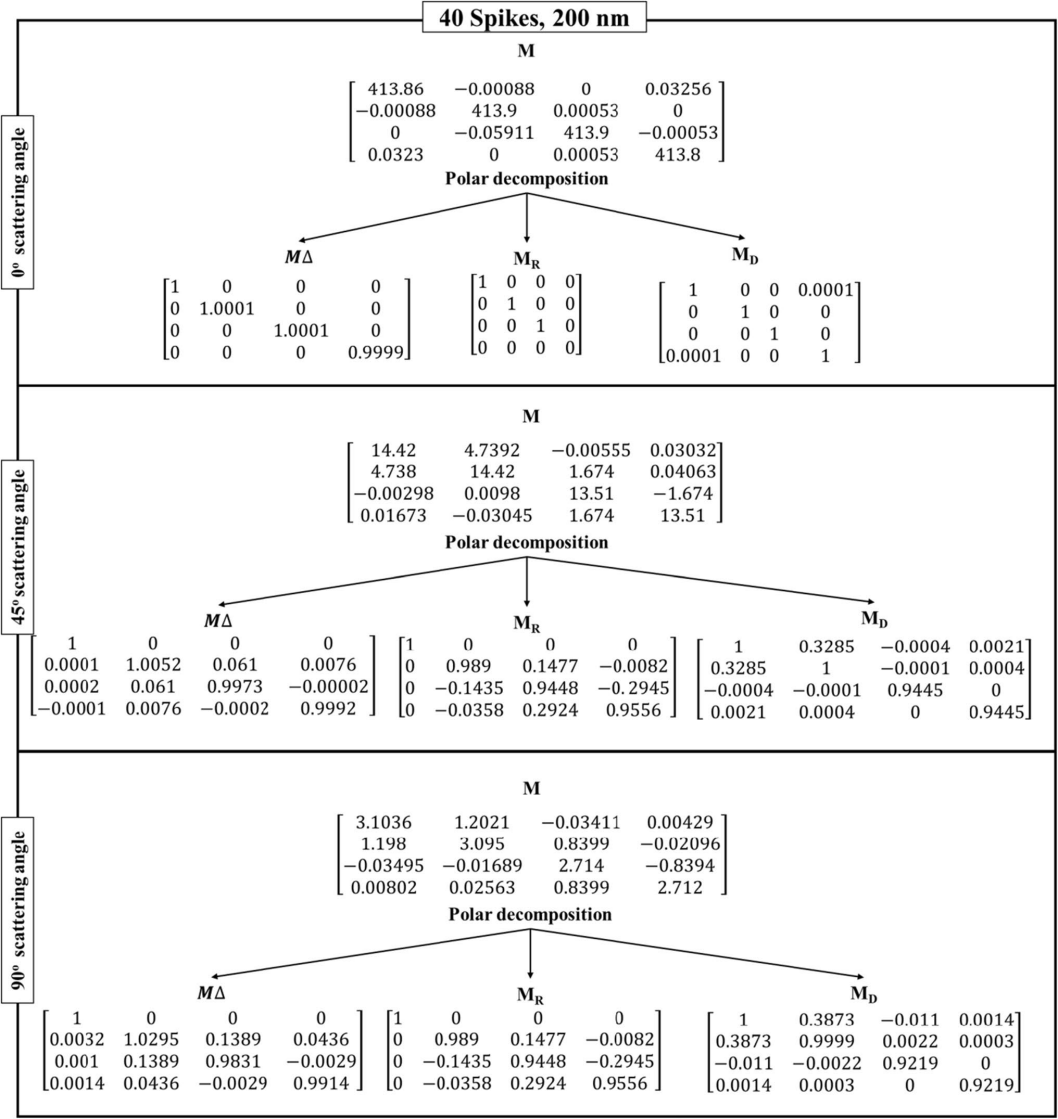
 
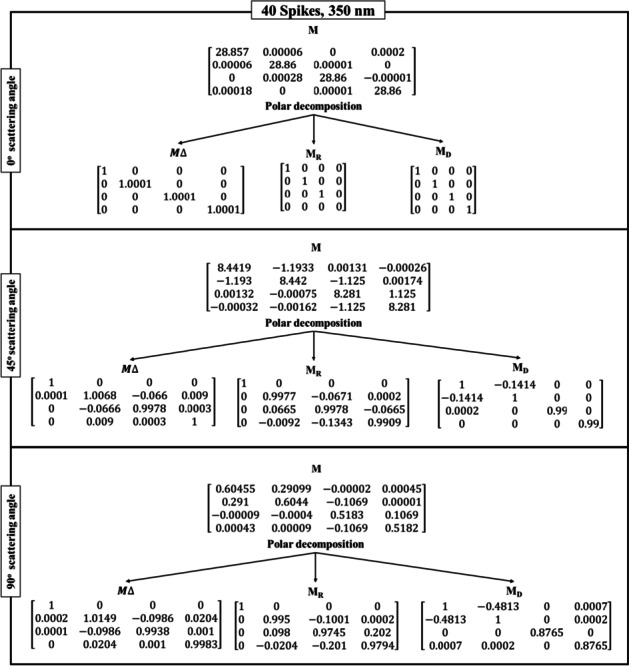


**Fig. 4:**
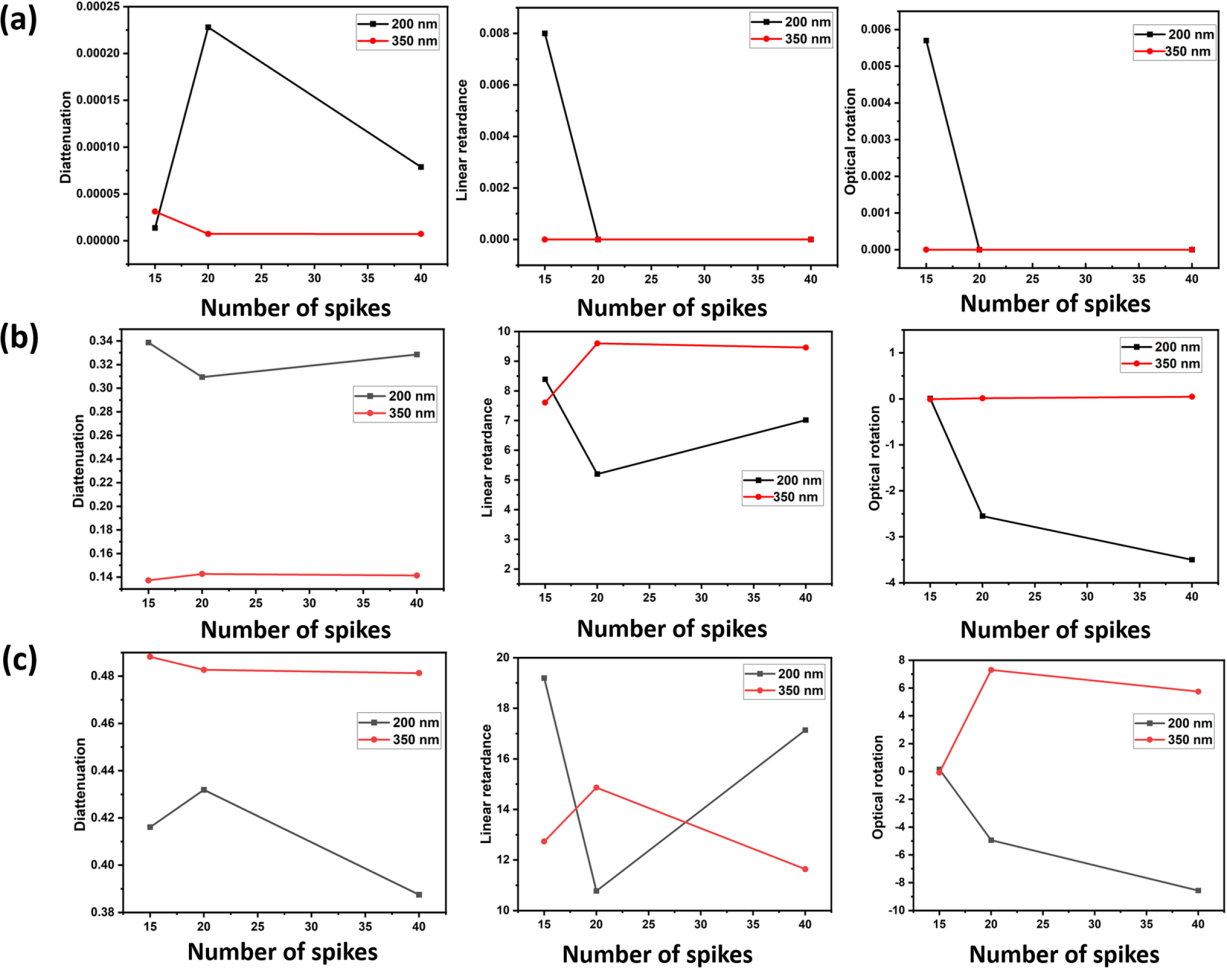
Graph of Lu–Chipman decomposition parameters such as diattenuation (*d*), linear retardance (*δ*), and optical rotation (*ψ*), as a function of spikes number at different wavelengths 200 nm and 350 nm for (**a**) 0°, (**b**) 45°, and (**c**) 90° scattering angle

Table 3 shows the Lu–Chipman decomposition matrices, such as depolarization ($${M}_{\Delta })$$, retardance ($${M}_{R})$$, and diattenuation ($${M}_{D})$$ of the SARS-CoV-2 models at 0°, 45°, and 90° scattering angles for both 200 nm and 350 nm incident wavelengths. It can be observed that all the spike models yielded negative depolarization (*∆*) values, prominently at 0°, 45°, and 90° scattering angles for both incident wavelengths. At 0° and 45° scattering angles, all the models were found to exhibit greater diattenuation (*d*) value for 200 nm incident wavelength, whereas, at 90° scattering angle, a higher diattenuation value was observed at 350 nm for all the spike numbers. Further, the diattenuation value was observed to be the highest (≥ 1) at 90° scattering angle at 350 nm. For the 15-spike model, a negative optical rotation (*ψ*) was observed for both 45° and 90° scattering angles at 350 nm incident wavelength; however, in case of the models with 20 and 40 spikes, the negative optical rotation was observed at 200 nm incident wavelength. The Lu–Chipman decomposition parameters such as diattenuation (*d*), depolarization (*∆*), retardance (*R*), optical rotation (*ψ*), and linear retardance (*δ*) for spike models (15, 20, and 40 spikes) at various scattering angles (0°, 45°, and 90°), at two different wavelengths (200 nm and 350 nm) are given in Table 3. The graph of Lu–Chipman decomposition parameters such as diattenuation (*d*), linear retardance (*δ*), and optical rotation (*ψ*) as a function of spike number at 200 nm and 350 nm for 0°, 45°, and 90° scattering angle is shown in Fig. 4.

**Table 3:** Lu–Chipman decomposition parameters

Spikes	Wavelength	Scattering angle (*θ*)	Diattenuation (*d*)	Depolarization (*∆*)	Retardance (*R*)		Optical rotation (*ψ*)	Linear retardance (*δ*)	
					Degree	Radian		Degree	Radian
15	200 nm	0°	1.37E − 05	− 3.33E − 09	0.0057	9.94E − 05	0.0057	0.008i	0.00013
		45°	0.3387	2.60E − 03	8.3857	0.1463	0.0086	8.3891	0.1464
		90°	0.4161	0.0167	19.1904	0.3349	0.1503	19.1947	0.335
	350 nm	0°	3.12E-05	− 1E − 04	0	0	0	0	0
		45°	0.1373	3.27E − 05	7.5856	0.132	− 0.0086	7.6067	0.132
		90°	0.4882	0.0053	12.7397	0.222	-0.0899	12.7344	0.222
20	200 nm	0°	2.28E − 04	1E − 04	0	0	0	0	0
		45°	0.3094	1.6E − 03	5.8088	0.101	− 2.55	5.2	0.09
		90°	0.4319	0.018	11.8583	0.206	− 4.9427	10.7741	0.186
	350 nm	0°	7.27E − 06	0	0	0	0	0	0
		45°	0.1427	− 1.70E − 03	8.7671	0.152	0.0144	9.5986	0.16
		90°	0.4827	− 6.8E − 04	16.5463	0.288	7.3006	14.8628	0.244
40	200 nm	0°	7.87E − 05	− 3.33E − 05	0.0029	5.06E − 05	0	0	0
		45°	0.3285	− 5.77E − 04	7.892	0.137	− 3.4992	7.018	0.124
		90°	0.3875	− 0.0013	19.1391	0.334	− 8.5635	17.137	0.299
	350 nm	0°	7.24E − 06	− 1E-04	0	0	0	0	0
		45°	0.1414	− 1.5E − 03	8.6389	0.15	0.0462	9.46	0.165
		90°	0.4813	− 2.30E − 03	12.9879	0.226	5.7437	11.6391	0.203

As the scattering angle increases, decomposition parameter values such as diattenuation and linear retardance are found to exhibit a gradual increase in their values, irrespective of the number of spikes in the SARS-CoV-2 model. At the scattering angle of 0°, all the models show variation in diattenuation values for 200 nm incident wavelength, whereas diattenuation was found to be 0 for 350 nm incident wavelength. Further, at 45° scattering angle, the sample exhibits diattenuation and retardance values for both 200 nm and 350 nm but does not exhibit any optical rotation for 350 nm. For 90° scattering angle, the sample shows variation in every decomposition parameter irrespective of the spike model and incident wavelength.

## Conclusion

During the past two years, humankind has witnessed unprecedented threat from SARS-CoV-2 that caused millions of deaths and adversely modulated lives and livelihoods throughout the world. The spike proteins of SARS-CoV-2 is the key of the virus that enables it to bind and invade human cells, and the virus infectivity increases significantly with a larger number of spike proteins. In this work, we have carried out light scattering calculations on SARS-CoV-2 models with 15, 20, and 40 numbers of spike proteins on the viral capsid surface to investigate how these numbers affect the light scattering properties by using DDSCAT 7.3.0 software package based on discrete dipole approximation. Notably, significant variations in the scattering properties were observed for all the three target geometries compared to that of a solid sphere of equal size, suggesting that the number of spikes is an essential parameter to understand the interaction of light with such virus particles. Further, the SARS-CoV-2 3D model assessed through Stokes polarimetry and Lu–Chipman decomposition approach revealed the individual polarization properties such as DOP, DOLP, and DOCP. Remarkably, the coronavirus models display higher DOP and DOLP values and vanishingly small DOCP values for 0° input polarization irrespective of their spike numbers. This indicates the presence of chiral/helical structures in the SARS-CoV-2. However, the models follow reverse trend for RCP input by exhibiting greater DOP and DOCP values but negligible DOLP values. This observation is critical as it indicates the capability of light scattering techniques to distinguish viral RNA, a chiral genetic structure. On the other hand, Lu–Chipman-based Mueller matrix polar decomposition method was employed to obtain various polarization properties of samples. Stokes–Mueller polarimetry-based characterization can provide insightful information of virions in a remote sensing architecture.

### Supplementary Information

Below is the link to the electronic supplementary material.

Supplementary file1 (DOCX 380 KB)

## Data Availability

Data will be available on request to the corresponding author.
