# Supplementary material for: Stokes–Mueller polarization-based analysis of model SARS-CoV-2 virions
Source: Lasers Med Sci. 2023 Jan 9;38(1):35. doi: 10.1007/s10103-022-03680-3 (PMC9827445; doi:10.1007/s10103-022-03680-3)
Supplement: Supplementary file 1 — Supplementary file1 (DOCX 380 KB) [file 10103_2022_3680_MOESM1_ESM.docx]

**Stokes-Mueller polarization based analysis of model SARS-CoV-2 virions**

**
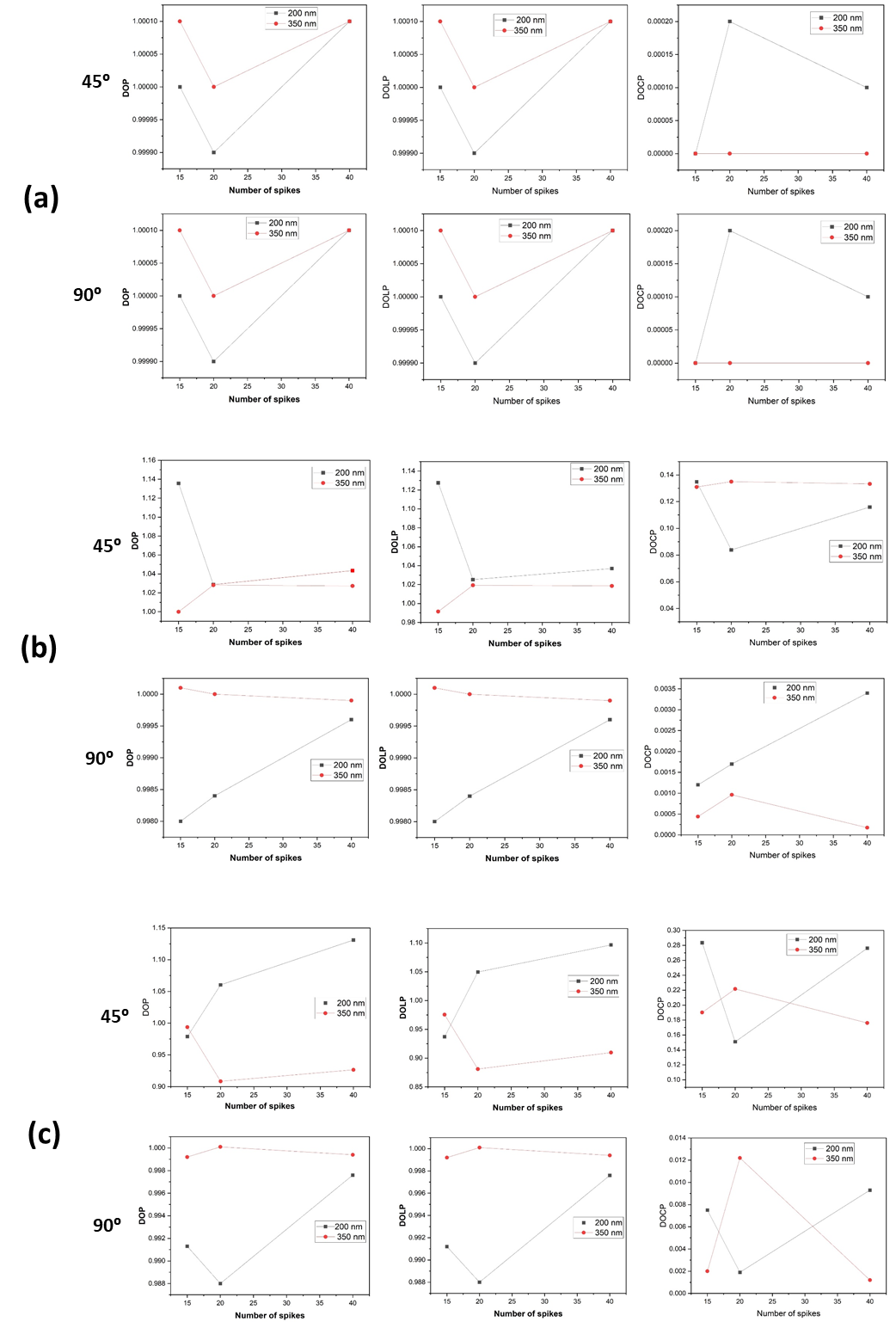
**

Figure 1. Graphs of Stokes polarization parameters as a function of spike number with 45° and 90° input polarization states at two wavelengths 200 nm and 350 nm for (a) 0⁰ (b) 45⁰, (c) 90⁰ scattering angles.
